# Differences in the effectiveness of leukocyte-rich platelet-rich plasma compared with leukocyte-poor platelet-rich plasma in the treatment of rotator cuff surgery: an umbrella review of meta-analyses

**DOI:** 10.1186/s10195-024-00791-1

**Published:** 2024-10-24

**Authors:** Peiyuan Tang, Masoud Rahmati, Wenfeng Xiao, Ting Wen, Dong Keon Yon, Lee Smith, Jingyue Su, Shengwu Yang, Yusheng Li, Zhenhan Deng

**Affiliations:** 1 Department of Orthopedics, Xiangya Hospital, Central South University, Changsha, 410008 China; 2 Department of Orthopaedic Surgery, The First Affiliated Hospital of Wenzhou Medical University, Wenzhou, 325000 China; 3 National Clinical Research Center for Geriatric Disorders, Xiangya Hospital, Central South University, Changsha, 410008 China; 4 Department of Physical Education and Sport Sciences, Faculty of Literature and Human Sciences, Lorestan University, Khoramabad, Iran; 5 Department of Physical Education and Sport Sciences, Faculty of Literature and Humanities, Vali-E-Asr University of Rafsanjan, Rafsanjan, Iran; 6 Department of Regulatory Science, Kyung Hee University, Seoul, South Korea; 7 Department of Pediatrics, Kyung Hee University Medical Center, Kyung Hee University College of Medicine, Seoul, South Korea; 8 Centre for Health, Performance, and Wellbeing, Anglia Ruskin University, Cambridge, UK

**Keywords:** Rotator cuff, Platelet-rich plasma, Umbrella review, Meta-analyses

## Abstract

**Background:**

An umbrella review of meta-analyses was conducted to evaluate the use of platelet-rich plasma (PRP) in arthroscopic surgeries of rotator cuff injury. The effectiveness of leukocyte-poor PRP and leukocyte-rich PRP in the treatment of rotator cuff surgery was also compared.

**Methods:**

Web of Science, Embase, PubMed/MEDLINE, and the Cochrane Library were searched from inception to May 2024. Literature screening, quality evaluation, and data extraction were performed according to the inclusion and exclusion criteria. The Jadad decision algorithm was used to ascertain which meta-analysis represented the best evidence.

**Results:**

A total of 11 meta-analyses with evidence level ranging from level 1 to 2 were included in this umbrella review. Leukocyte-poor PRP was effective in reducing rotator cuff retear rates, alleviating pain, and increasing Constant scores compared with non-PRP treatments. However, it did not show improvement on the University of California Los Angeles (UCLA) score, the American Shoulder and Elbow Surgeons (ASES) score, and the Simple Shoulder Test (SST) compared with the non-PRP treatment group. Meanwhile, the leukocyte-rich PRP group improved the SST but showed no different results when compared with the non-PRP treatment group.

**Conclusion:**

Compared with no use of PRP, leukocyte-poor PRP was able to alleviate postoperative pain, reduce the retear rate, and improve the postoperative Constant score. Leukocyte-rich PRP could effectively enhance postoperative SST outcomes, leading to improvement of patient satisfaction and quality of life. Future researches should prioritize long-term follow-up studies and evaluate the durability of these results.

**Supplementary Information:**

The online version contains supplementary material available at 10.1186/s10195-024-00791-1.

## Introduction

The most common cause of rotator cuff injuries is degenerative or senescent change with aging [1]. More than 50% of patients aged over 80 years old suffer from pain and weakness caused by rotator cuff injuries [2, 3] that substantially hinder their daily activities, affecting their work, physical activity, and sleep [4]. Untreated rotator cuff injuries may eventually result in osteoarthritis and other degenerative changes of the shoulder [5]. Traditional therapies include acupuncture, massage, topical herbal medicine, bone marrow aspirate concentrate, and so on, aiming to promote tissue repair by providing growth factors and structural support. Although they have shown some efficacy, their effectiveness and safety require further investigation. Platelet-rich plasma (PRP), derived from autologous whole blood and boasting an elevated concentration of platelets, has gained increasing attention due to its simple preparation and high safety profile [6, 7].

PRP can be utilized with sutures and repair materials to facilitate the healing and restoration of the tendons in arthroscopic surgeries [8]. According to the content of white blood cells (WBCs), PRP can be divided into leukocyte-poor PRP and leukocyte-rich PRP [9]. Leukocyte-poor PRP reduces the amount of WBCs during preparation, while the number of WBCs is higher in leukocyte-rich PRP. Due to their different biological activities, leukocyte-poor PRP is more suitable for situations that reduce inflammatory response, and leukocyte-rich PRP is often used in situations that require a strong inflammatory response to promote healing and repair [10]. There is no consensus on PRP’s effectiveness in the treatment of rotator cuff injuries yet. The purpose of this study is to conduct an umbrella review of meta-analyses (MAs) to evaluate the use of PRP in the arthroscopic surgery treatment of rotator cuff injuries, and the effects of leukocyte-poor PRP and leukocyte-rich PRP on rotator cuff injury were also compared, to provide an accurate and comprehensive understand of the advantages and drawbacks of this treatment.

## Methods

An umbrella review evaluating and compiling data from various MAs on all outcomes was performed [11, 12], following the Cochrane Handbook's guidelines [12–15]. Registered on the PROSPERO website, the report on the work complied with A Measurement Tool to Assess systematic Reviews 2 (AMSTAR 2) and Preferred Reporting Items for Systematic Reviews and Meta-Analyses (PRISMA) [16, 17]. (Supplementary Material S1) The procedures of data retrieval, extraction, processing, and evaluation were delegated to two independent researchers. A third reviewer acted as a judge if there was any disagreement [18, 19].

### Search strategy

Four databases including Web of Science, Embase, PubMed/MEDLINE, and the Cochrane Library were searched from inception to May 2024. The English search terms included platelet-rich plasma, platelet rich plasma, plasma, platelet-rich, rotator cuff, cuff, rotator [20], rotator cuffs, systematic review, meta-analyses, etc. (Supplementary Material S2). The mesh terms can be found in Supplementary Material S6.

### Screening and selection of MAs

One researcher checked the titles and abstracts to screen any unnecessary articles. The remaining studies’ full text was located and evaluated separately by two researchers. The MAs that were included should meet the subsequent inclusion criteria [21]: (1) MAs should be defined by the PRISMA [12, 22] with interventions including leukocyte-rich PRP or leukocyte-poor PRP; (2) outcome measurements for MA should include at least two trials, and must be quantitatively synthesized and evaluate either effect or safety; (3) there should be sufficient extractable data, such as effect sizes, 95% confidence intervals (CIs), and the number of included studies for each outcome. The exclusion criteria were as follows: (1) letters, conference abstracts, and protocols; (2) MAs that lack sufficient extractable information about PRP, for instance, the quantity of pooled trials, and the 95% CI for the relative effect [18, 23]; (3) MAs focusing on other types of PRP or other biologic therapies.

### Extraction of data and evaluation of quality

The data were extracted independently by two independent researchers, who then assessed their quality, while a third person helped to reach a consensus on any differences. The primary outcomes included retear rates, Constant score, University of California, Los Angeles (UCLA) score, visual analog scale (VAS), the American Shoulder and Elbow Surgeons (ASES) score, and the Simple Shoulder Test (SST) [24]. Additionally, the year of publication and the number of randomized control trials (RCTs) included in each MA were extracted. Information on the type of PRP, sex ratio, mean age, number of patients in both the PRP and non-PRP groups, and the level of evidence was also collected. Using the AMSTAR 2, two researchers assessed the included MAs’ methodological quality independently. When a dispute arose, it was reevaluated by the third researcher to reach a consensus [12, 16].

### Select the best evidence

**Fig. 1:**
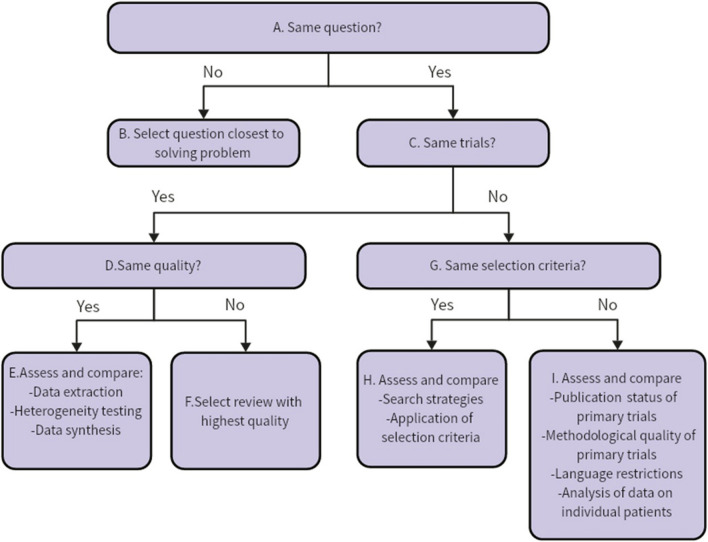
Jadad decision algorithm

The Jadad decision algorithm was employed to provide recommendations for the best evidence and identify sources of inconsistency between MAs [25] (Fig. 1). The algorithm consisted of three main steps: (1) assessing the methodological quality of the included MAs using a validated tool, such as AMSTAR 2; (2) comparing the publication dates of the included MAs, with the most recent MA generally considered to provide the best available evidence; (3) evaluating the comprehensiveness of the literature search performed in each MA, with more extensive and inclusive search strategies considered to provide more reliable evidence. The tool was used independently by the two researchers to determine which MAs offered the greatest evidence at the time. The Jadad decision algorithm was not applied if all MAs had addressed the same research objectives and yielded similar results.

## Results

### Search results

**Fig. 2:**
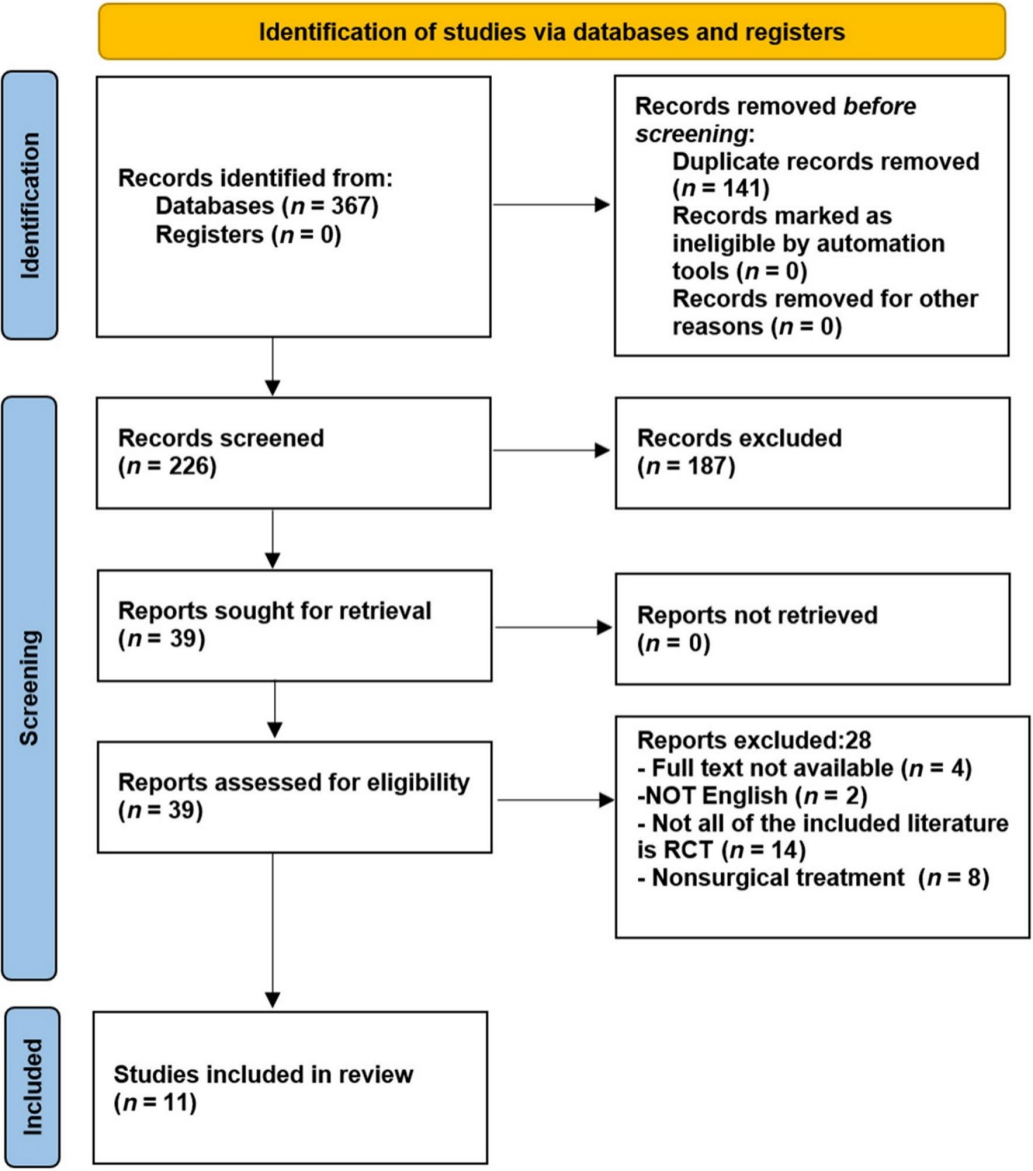
The Preferred Reporting Items for Systematic reviews and Meta-analysis (PRISMA) flow diagram to show study selection

The search strategy yielded 367 articles in total, of which 141 were eliminated after removing duplicates. Subsequently, 39 articles were excluded after a strict review of titles and abstracts [26–28]. After full text review, 28 more studies were excluded (Supplementary Material S3); thus, 11 studies were included finally (Fig. 2).

### The assessment of MAs

The AMSTAR 2 was applied to evaluate system reviews. It assessed aspects such as program registration, comprehensive literature search, and bias risk assessment, ensuring that the most critical methodological elements were reported. All included studies had an AMSTAR-2 rating of moderate to high quality (Supplementary Material S4).

### Study characteristics

The present review consisted of MAs published between 2013 and 2023. All of the MAs used in the analysis had RCTs as their foundation. Five studies were listed as evidence level 1, and the remaining were level 2 (Table 1). Eight studies presented clear sex ratios [3, 27, 29–34]. Only one study did not present the number of patients in the PRP and non-PRP groups [29]. Two studies did not provide a clear PRP type, and the remaining study provided the PRP types [29, 32].

**Table 1:** Baseline characteristics of included literature

Study	Year	Number of RCTs included	PRP type	Sex ratio		Mean age		Number of patients		Level of evidence
				Man	Woman	PRP	Non-PRP	PRP	Non-PRP	
Feltri [1]	2023	18	PRP	991	1190	54.3	54.3	NA	NA	I
Li [2]	2022	23	LP; LR	665	719	58.9	58.7	709	731	II
Zhao [3]	2021	10	LP	313	429	58.2	57.7	372	370	II
Xu [4]	2021	14	LP; LR	423	500	58.5	58.5	458	465	I
Ryan [5]	2021	17	LP; LR	NA	NA	NA	NA	553	551	II
Yang [6]	2020	7	LP; LR	NA	NA	57.6	57.5	273	268	II
Chen [7]	2020	17	LP; LR	529	622	58.2	58.2	545	624	I
Han [8]	2019	13	LP; LR	418	462	58.7	58.5	439	441	II
Wang [9]	2019	8	PRP	250	306	58.9	58.4	283	283	I
Zhao [10]	2015	8	LP; LR	232	232	60.1	60.2	234	230	II
Cai [11]	2015	5	LP; LR	NA	NA	59.2	59.04	150	153	I

NA, not available; PRP, platelet-rich plasma; LP, leukocyte-poor; LR, leukocyte-rich

This review also covered and integrated all the conclusions from the literature. PRP did not affect the surgical treatment results of rotator cuff injuries [34]. Two MAs suggested that PRP application could promote postoperative healing of the rotator cuff injuries [27, 35]. Seven MAs showed that PRP administration is able to reduce the retear rates after rotator cuff surgeries [3, 29–33, 36]. Two MAs suggested that PRP treatment may be beneficial for small- to medium-size rotator cuff injuries [37, 38].

### Results of MAs

#### Retear rates

A total of five MAs involved retear rates [3, 27, 30, 34, 35]. They were divided into the leukocyte-rich PRP group and the leukocyte-poor PRP group. Leukocyte-poor PRP showed significant reduction in retear rates compared with non-PRP treatments in studies by Li et al. [3], Zhao et al. [27], Yang et al. [35], and Xu et al. [30] However, Zhao et al. [34] found no statistically significant difference between the leukocyte-poor PRP and the non-PRP treatment groups. (Supplementary Material S5-Table 1) Meanwhile, the leukocyte-rich PRP group did not show any significant difference in reducing retear rates compared with the non-PRP treatment group. (Supplementary Material S5 Table 7) Based on the Jadad decision algorithm, it was determined that the study by Li et al. [3] represents the best available evidence. The results showed that leukocyte-poor PRP was able to reduce the postoperative retear rates, while leukocyte-rich PRP could not, compared with non-PRP treatments.

#### Constant score

7 MAs reported Constant scores in total [3, 27, 33–36, 38]. Leukocyte-poor PRP significantly increased the Constant score compared with non-PRP treatments in studies by Li et al. [3], Zhao et al. [27], and Han et al. [33] However, Ryan et al. [36], Yang et al. [35], Zhao et al. [34], and Cai et al. [38] found no significant difference between the leukocyte-poor PRP and non-PRP treatment groups. (Supplementary Material S5 Table 2) Meanwhile, leukocyte-rich PRP significantly increased the Constant scores compared with non-PRP treatments in studies by Ryan et al. [36] and Han et al. [33] However, Li et al. [3] found no statistically significant difference between the leukocyte-rich PRP and non-PRP treatment groups. (Supplementary Material S5 Table 8) Based on the Jadad decision algorithm, it was concluded that the study by Li et al. [3] represented the best available evidence. The results showed that leukocyte-poor PRP improved the postoperative Constant scores, while leukocyte-rich PRP did not, compared with non-PRP treatments.

#### University of California, Los Angeles score

Seven MAs included UCLA scores [3, 27, 33–36, 38]. The leukocyte-poor PRP group significantly increased the UCLA scores compared with the non-PRP treatment group in a study by Zhao et al. [27]. However, Li et al. [3], Ryan et al. [36], Yang et al. [35], Zhao et al. [34], Cai et al. [38], and Han et al. [33] found no statistically significant difference between the leukocyte-poor PRP group and non-PRP group. (Supplementary Material S5 Table 3) Based on the Jadad decision algorithm, it was concluded that the study by Li et al. [3] represented the best available evidence. The results showed that leukocyte-poor PRP did not improve the UCLA score compared with no PRP treatments.

#### Visual analog scale

VAS was reported in five MAs [3, 27, 33, 35, 36]. The leukocyte-poor PRP group reduced the postoperative VAS compared with the non-PRP treatment group (Supplementary Material S5 Table 4).

#### American Shoulder and Elbow Surgeons score

Five MAs included ASES scores [3, 27, 33, 36, 38]. The leukocyte-poor PRP group did not improve the postoperative ASES scores compared with the non-PRP treatment group (Supplementary material S5 Table 5).

#### Simple Shoulder Test

Only two MAs reported SST [33, 36]. Leukocyte-poor PRP did not improve the postoperative SST, while leukocyte-rich PRP improved the postoperative SST, compared with non-PRP treatments (Supplementary Material S5 Tables 6 and 9).

## Discussion

The most importing finding of the present study was that leukocyte-poor PRP was able to relieve postoperative pain, reduce retear rates, and improve postoperative Constant scores, while leukocyte-rich PRP only improved postoperative SST. The different effects of these two types of PRP may be attributed to the unique composition and biological characteristics of its formulation. The superior analgesic effect of leukocyte-poor PRP could be explained by its low concentration of proinflammatory cytokines. A reduced inflammatory response might contribute to a more comfortable recovery process. Additionally, the lower WBC content in leukocyte-poor PRP may provide a more favorable environment for tissue healing, which may explain the reduced tear rates. The higher WBC content in leukocyte-rich PRP may stimulate a stronger healing response by releasing extra growth factors and cytokines, which can improve tissue repair and healing and result in higher SST.

Among the MAs included in this umbrella review, some results differed from those identified as the best evidence by the Jadad decision algorithm. The results of five studies on retear rates in the leukocyte-poor PRP group were inconsistent, but the conclusions by Zhao et al. [34] were inconsistent with others. Zhao et al. [34] founded that there was no statistical difference between the leukocyte-poor PRP group and the non-PRP treatment group. The differences in the initial disease severity, age, sex ratio, and other demographic characteristics of patients in the two studies influence the treatment effect. The differences in the distribution of these factors in different studies led to inconsistent results of MA. The results of seven studies on UCLA scores in the leukocyte-poor PRP group were inconsistent, and Zhao et al. [27] concluded that leukocyte-poor PRP could improve the UCLA score of patients with rotator cuff surgery, which was inconsistent with other studies. The two studies used different methods of randomization, blinding implementation, and patient grouping criteria, and these design differences might have resulted in the different outcomes. The results of seven studies on Constant scores in the leukocyte-poor PRP group were inconsistent. The conclusions drawn by Ryan et al. [36], Yang et al. [35], Zhao et al. [34], and Cai et al. [38] are at odds with the best evidence. They suggested that leukocyte-poor PRP could not improve Constant scores in patients underwent rotator cuff surgery. The reason for the inconsistent results of Yang et al. [35], Zhao et al. [34], and Cai et al. [38] were the small number of studies included. The inconsistent results in Ryan et al. [36] may be attributed to the low-quality studies included. The results of three studies reporting Constant scores in the the leukocyte-rich PRP group were inconsistent. Inconsistent with the best evidence, Ryan et al. [36] and Han et al. [33] concluded that leukocyte-rich PRP could improve Constant scores in patients undergoing rotator cuff repair surgeries. This may due to the inconsistent follow-up times in the studies included by Ryan et al. [36] and Han et al. [33], which led to conclusions inconsistent with the best evidence. Differences in the follow-up length may also affect the long-term results. Some studies may observe positive effects over shorter follow-up periods, while longer follow-up may reveal different results.

When comparing our results with previous publications, Lv et al. [39] suggested that arthroscopic PRP treatment can reduce postoperative pain and improve Constant scores scores. Jiang et al. [40] showed that PRP is helpful in relieving the long-term pain of rotator cuff tears. These results are consistent with our findings despite no mention of PRP type. However, Saltzman BM et al. showed that PRP is not usually used to improve clinical outcome scores or reduce retear rates in arthroscopic rotator cuff repair procedures [41, 42], which is contrary to the conclusion of our study. It was noted that the literature included in this MA was of average quality and objective statistical measurements were not used in data analysis. In addition, Cavendish et al. [43] suggested that the effectiveness of PRP could vary depending on its dosage form, making it difficult to provide a specific recommendation in the treatment of rotator cuff repair.

This umbrella review has several limitations. Firstly, the included MAs may possess inherent selection, reporting, and publication biases, which could potentially influence the overall findings. Secondly, many primary studies did not provide detailed information on follow-up or specific outcome measurements, which may limit the comparability of results across studies. Thirdly, the rapid publication of new high-quality RCTs in this field means that the currently available evidence may need to be updated frequently to incorporate the latest findings. It is also important to note that this umbrella review included only MAs published in English, excluding potentially relevant studies published in other languages. Moreover, many of the included MAs focused on specific patient populations, regions, or conditions, which may limit the generalizability of the findings to a broader population. Lastly, the variability in study quality and the different levels of evidence among the included MAs may have implications for the reliability and robustness of the conclusions drawn from this umbrella review.

## Conclusion

Compared with no use of PRP, leukocyte-poor PRP was proven to be able to alleviate postoperative pain, reduce the retear rate, and improve the postoperative Constant score. Leukocyte-rich PRP could effectively enhance postoperative SST outcomes, ultimately leading to improvement of patient satisfaction and quality of life. Future research should prioritize long-term follow-up studies and evaluate the durability of these results.

## Supplementary Information

Additional file 1.

Additional file 2.

Additional file 3.

Additional file 4.

Additional file 5.

Additional file 6.

## Data Availability

Three databases, that is, PubMed, Embase, and Web of Science, were searched up to September 2023. The data are publicly available, and there are no restrictions. The data from this study can be shared with other researchers. The inconsistencies in results between meta-analysis make it difficult for clinicians to select specific treatment measures. To assist clinicians in selecting among inconsistent reviews, we employed the Jadad decision algorithm to provide recommendations for best evidence. The Jadad decision algorithm identifies sources of inconsistency between meta-analyses, and it has been frequently utilized to provide therapy suggestions based on disagreement in meta-analysis outcomes. The tool was used independently by the two writers, who then agreed on which meta-analysis offered the greatest evidence at the time. We would not apply the Jadad choice algorithm if all meta-analyses of OA medications took into account the same research objectives and produced similar results. The data in this study comply with applicable legal, ethical, and privacy regulations. The data in this study are reproducible and robust.

## Abbreviations

AMSTAR 2: A Measurement Tool to Assess systematic Reviews 2
ASES: American Shoulder and Elbow Surgeons score
CIs: Confidence intervals
MA: Meta-analysis
PRISMA: Preferred Reporting Items for Systematic Reviews and Meta-Analyses
PRP: Platelet-rich plasma
RCTs: Randomized control trials
SST: Simple shoulder test
UCLA: University of California, Los Angeles
VAS: Visual analog scale
WBCs: White blood cells
