# Supplementary material for: Differences in the effectiveness of leukocyte-rich platelet-rich plasma compared with leukocyte-poor platelet-rich plasma in the treatment of rotator cuff surgery: an umbrella review of meta-analyses
Source: J Orthop Traumatol. 2024 Oct 24;25:50. doi: 10.1186/s10195-024-00791-1 (PMC11502652; doi:10.1186/s10195-024-00791-1)
Supplement: Supplementary file 2 — Additional file 2. [file 10195_2024_791_MOESM2_ESM.docx]

# 1.

Pubmed

Search: ((("Platelet-Rich Plasma"[Mesh]) OR (((Plasma, Platelet-Rich[Title/Abstract]) OR (Platelet Rich Plasma[Title/Abstract])) OR (Platelet-rich Plasma[Title/Abstract]))) AND ((((((((Rotator Cuff[Title/Abstract]) OR (Cuff, Rotator[Title/Abstract])) OR (Rotator Cuffs[Title/Abstract])) OR (Teres Minor[Title/Abstract])) OR (Subscapularis[Title/Abstract])) OR (Infraspinatus[Title/Abstract])) OR (Supraspinatus[Title/Abstract])) OR ("Rotator Cuff"[Mesh]))) AND ((((Meta-analysis[Title/Abstract]) OR ("Meta-Analysis" [Publication Type])) OR (Systematic review[Title/Abstract])) OR ("Systematic Review" [Publication Type])) Sort by: Publication Date

2023/8/11

Results:86

# 2.

## Embase

No. Query Results Results

#12. #9 AND #10 AND #11 128

#11. #1 OR #5 23,676

#10. #2 OR #6 27,337

#9. #3 OR #4 OR #7 OR #8 701,631

#8. 'meta analysis':ab,ti 300,034

#7. 'systematic review':ab,ti 325,887

#6. 'rotator cuff':ab,ti OR 'cuff, rotator':ab,ti OR 22,635

'rotator cuffs':ab,ti OR 'teres minor':ab,ti OR

subscapularis:ab,ti OR infraspinatus:ab,ti OR

supraspinatus:ab,ti

#5. 'plasma, platelet-rich':ab,ti OR 'platelet rich 17,474

plasma':ab,ti OR 'platelet-rich plasma':ab,ti

#4. 'meta analysis'/exp OR 'meta analysis' 413,168

#3. 'systematic review'/exp OR 'systematic review' 529,500

#2. 'rotator cuff'/exp OR 'rotator cuff' 24,601

#1. 'thrombocyte rich plasma'/exp OR 'thrombocyte 19,289

rich plasma'

2023/8/11

Results:128

# 3.

## Web of science

1: ((TS=(Platelet-rich Plasma)) OR TS=(Plasma, Platelet-Rich)) OR TS=(Platelet Rich Plasma) and Preprint Citation Index

2: ((((((TS=(Rotator Cuff)) OR TS=(Cuff, Rotator)) OR TS=(Rotator Cuffs)) OR TS=(Teres Minor)) OR TS=(Subscapularis)) OR TS=(Infraspinatus)) OR TS=(Supraspinatus) and Preprint Citation Index

3: (TS=(Systematic review)) OR TS=(Meta-analysis) and Preprint Citation Index

4: #3 AND #2 AND #1 and Preprint Citation Index

2023/8/11

Results:151

# 4.

## Cochrane

#1 ("meta analysis"):ti,ab,kw OR ("systematic review"):ti,ab,kw 24781

#2 (Supraspinatus):ti,ab,kw OR (Rotator Cuff):ti,ab,kw 2833

#3 (Cuff, Rotator):ti,ab,kw OR (Rotator Cuffs):ti,ab,kw OR (Teres Minor):ti,ab,kw OR (Subscapularis):ti,ab,kw OR (Infraspinatus):ti,ab,kw 2801

#4 MeSH descriptor: [Rotator Cuff] explode all trees 572

#5 MeSH descriptor: [Platelet-Rich Plasma] explode all trees 944

#6 (Plasma, Platelet-Rich):ti,ab,kw OR (Platelet Rich Plasma):ti,ab,kw OR (Platelet-rich Plasma):ti,ab,kw 3120

#7 #6 OR #5 3319

#8 #4 OR #3 OR #2 3022

#9 #7 AND #8 AND #1 2

2023/8/11

Results:2
