# Supplementary material for: Differences in the effectiveness of leukocyte-rich platelet-rich plasma compared with leukocyte-poor platelet-rich plasma in the treatment of rotator cuff surgery: an umbrella review of meta-analyses
Source: J Orthop Traumatol. 2024 Oct 24;25:50. doi: 10.1186/s10195-024-00791-1 (PMC11502652; doi:10.1186/s10195-024-00791-1)
Supplement: Supplementary file 3 — Additional file 3. [file 10195_2024_791_MOESM3_ESM.docx]

| Study | - Reason for exclusion |
| --- | --- |
| [[1](#_ENREF_1)] | - Full text not available |
| [[2](#_ENREF_2)] | - Full text not available |
| [[3](#_ENREF_3)] | - Full text not available |
| [[4](#_ENREF_4)] | - Full text not available |
| [[5](#_ENREF_5)] | - NOT English |
| [[6](#_ENREF_6)] | - NOT English |
| [[7](#_ENREF_7)] | - Not all of the included literature is RCTS |
| [[8](#_ENREF_8)] | - Not all of the included literature is RCTS |
| [[9](#_ENREF_9)] | - Not all of the included literature is RCTS |
| [[10](#_ENREF_10)] | - Not all of the included literature is RCTS |
| [[11](#_ENREF_11)] | - Not all of the included literature is RCTS |
| [[12](#_ENREF_12)] | - Not all of the included literature is RCTS |
| [[13](#_ENREF_13)] | - Not all of the included literature is RCTS |
| [[14](#_ENREF_14)] | - Not all of the included literature is RCTS |
| [[15](#_ENREF_15)] | - Not all of the included literature is RCTS |
| [[16](#_ENREF_16)] | - Not all of the included literature is RCTS |
| [[17](#_ENREF_17)] | - Not all of the included literature is RCTS |
| [[18](#_ENREF_18)] | - Not all of the included literature is RCTS |
| [[19](#_ENREF_19)] | - Not all of the included literature is RCTS |
| [[20](#_ENREF_20)] | - Not all of the included literature is RCTS |
| [[21](#_ENREF_21)] | - Nonsurgical treatment |
| [[22](#_ENREF_22)] | - Nonsurgical treatment |
| [[23](#_ENREF_23)] | - Nonsurgical treatment |
| [[24](#_ENREF_24)] | - Nonsurgical treatment |
| [[25](#_ENREF_25)] | - Nonsurgical treatment |
| [[26](#_ENREF_26)] | - Nonsurgical treatment |
| [[27](#_ENREF_27)] | - Nonsurgical treatment |
| [[28](#_ENREF_28)] | - Nonsurgical treatment |

## The study that was excluded after reading the full text and its related reasons:

# Reference:

1. Yang J, Sun Y, Xu P, Cheng B. Can patients get better clinical outcomes by using PRP in rotator cuff repair: a meta-analysis of randomized controlled trials. J. Sports Med. Phys. Fitness 2016;**56**(11):1359-67 [published Online First: 20151016].

2. Xiao W, Luo R, Sun J, et al. Efficacy and clinical outcomes of platelet-rich plasma for arthroscopic repair rotator cuff tears: a meta-analysis. Int. J. Clin. Exp. Med. 2016;**9**(10):19831-40.

3. Sanchez-Losilla C, Ferre-Aniorte A, Alvarez-Diaz P, Barastegui-Fernandez D, Cugat R, Alentorn-Geli E. Efficacy of platelet-rich plasma in rotator cuff repair: systematic review and meta-analysis. Revista espanola de cirugia ortopedica y traumatologia 2023 doi: 10.1016/j.recot.2023.05.014.

4. Lui M, Shih W, Yim N, Brandstater M, Ashfaq M, Tran D. Systematic Review and Meta-Analysis of Nonoperative Platelet-Rich Plasma Shoulder Injections for Rotator Cuff Pathology. Pm r 2021;**13**(10):1157-68 doi: 10.1002/pmrj.12516 [published Online First: 20210104].

5. Sun RJ, Guo L, Li PC, Wei XC. [Platelet-rich plasma vs corticosteroid for treatment of rotator cuff tendinopathy:a Meta-analysis]. Zhongguo Gu Shang 2022;**35**(12):1170-6 doi: 10.12200/j.issn.1003-0034.2022.12.012.

6. Zhang Q, Ge Ha, Zhou J, Cheng B. Are Platelet-Rich Products Necessary during the Arthroscopic Repair of Full-Thickness Rotator Cuff Tears: A Meta-Analysis. PLoS One 2013;**8**(7) doi: 10.1371/journal.pone.0069731.

7. Cavendish PA, Everhart JS, DiBartola AC, Eikenberry AD, Cvetanovich GL, Flanigan DC. The effect of perioperative platelet-rich plasma injections on postoperative failure rates following rotator cuff repair: a systematic review with meta-analysis. J. Shoulder Elbow Surg. 2020;**29**(5):1059-70 doi: 10.1016/j.jse.2020.01.084.

8. Weber SC, Parise C. Regarding "Efficacy of platelet-rich plasma in arthroscopic repair of full-thickness rotator cuff tears: a meta-analysis". J. Shoulder Elbow Surg. 2016;**25**(8):E246-E47 doi: 10.1016/j.jse.2016.04.032.

9. Trantos IA, Vasiliadis ES, Giannoulis FS, Pappa E, Kakridonis F, Pneumaticos SG. The Effect of PRP Augmentation of Arthroscopic Repairs of Shoulder Rotator Cuff Tears on Postoperative Clinical Scores and Retear Rates: A Systematic Review and Meta-Analysis. Journal of Clinical Medicine 2023;**12**(2) doi: 10.3390/jcm12020581.

10. Chahal J, Van Thiel GS, Mall N, et al. The role of platelet-rich plasma in arthroscopic rotator cuff repair: a systematic review with quantitative synthesis. Arthroscopy 2012;**28**(11):1718-27 doi: 10.1016/j.arthro.2012.03.007 [published Online First: 20120612].

11. Ahmad Z, Ang S, Rushton N, et al. Platelet-Rich Plasma Augmentation of Arthroscopic Rotator Cuff Repair Lowers Retear Rates and Improves Short-Term Postoperative Functional Outcome Scores: A Systematic Review of Meta-Analyses. Arthroscopy, sports medicine, and rehabilitation 2022;**4**(2):e823-e33 doi: 10.1016/j.asmr.2021.12.012.

12. Adra M, El Ghazal N, Nakanishi H, et al. Platelet-rich plasma versus corticosteroid injections in the management of patients with rotator cuff disease: A systematic review and meta-analysis. J. Orthop. Res. 2023;**41**(1):7-20 doi: 10.1002/jor.25463 [published Online First: 20221031].

13. Villarreal-Villarreal GA, Simental-Mendía M, Garza-Borjón AE, et al. Double-Row Rotator Cuff Repair Enhanced With Platelet-Rich Therapy Reduces Retear Rate: A Systematic Review and Meta-analysis of Randomized Controlled Trials. Arthroscopy 2021;**37**(6):1937-47.e1 doi: 10.1016/j.arthro.2020.12.191 [published Online First: 20201224].

14. Zhi F, Cai F, Zhang W, Xiong L, Hu J, Lin X. Clinical efficacy of different shoulder joint drug injections for rotator cuff injuries: A network meta-analysis. Medicine (Baltimore) 2022;**101**(39):e30659 doi: 10.1097/md.0000000000030659.

15. Tossolini Goulart L, Matsunaga FT, Belloti JC, Faloppa F, Paim TS, Tamaoki MJS. Effectiveness of subacromial injections in rotator cuff lesions: systematic review and meta-analysis protocol. Bmj Open 2022;**12**(11) doi: 10.1136/bmjopen-2022-062114.

16. Shen W, Wang W, Xue Y, Shi J, Pu Z, Gao Q. Comparative effectiveness of different platelet-rich plasma for arthroscopic rotator cuff repair: A protocol for systematic review and network meta-analysis. Medicine 2022;**101**(42) doi: 10.1097/md.0000000000031260.

17. Lv M, Xu Q, He F, et al. Transosseous-Equivalent/Suture Bridge Technique in Combination With Platelet-Rich Plasma Application Yield Optimal Clinical Outcomes in Arthroscopic Rotator Cuff Repair: A Bayesian Network Analysis of Randomized Controlled Trials. Arthroscopy 2023;**39**(2):425-37.e1 doi: 10.1016/j.arthro.2022.10.039 [published Online First: 20221105].

18. Lavoie-Gagne O, Fury MS, Mehta N, et al. Double-Row Repair With Platelet-Rich Plasma Optimizes Retear Rates After Small to Medium Full-Thickness Rotator Cuff Repair: A Systematic Review and Network Meta-analysis of Randomized Controlled Trials. Arthroscopy 2022;**38**(9):2714-29 doi: 10.1016/j.arthro.2022.03.014 [published Online First: 20220322].

19. Jiang X, Zhang H, Wu Q, Chen Y, Jiang T. Comparison of three common shoulder injections for rotator cuff tears: a systematic review and network meta-analysis. J. Orthop. Surg. Res. 2023;**18**(1):272 doi: 10.1186/s13018-023-03747-z [published Online First: 20230403].

20. Saltzman BM, Jain A, Campbell KA, et al. Does the Use of Platelet-Rich Plasma at the Time of Surgery Improve Clinical Outcomes in Arthroscopic Rotator Cuff Repair When Compared With Control Cohorts? A Systematic Review of Meta-analyses. Arthroscopy 2016;**32**(5):906-18 doi: 10.1016/j.arthro.2015.10.007 [published Online First: 20151223].

21. Zhu P, Wang Z, Li H, Cai Y. Platelet-Rich Plasma Injection in Non-Operative Treatment of Partial-Thickness Rotator Cuff Tears: A Systematic Review and Meta-Analysis. J. Rehabil. Med. 2022;**54**:jrm00312 doi: 10.2340/jrm.v54.1434 [published Online First: 20220916].

22. Xiang XN, Deng J, Liu Y, Yu X, Cheng B, He HC. Conservative treatment of partial-thickness rotator cuff tears and tendinopathy with platelet-rich plasma: A systematic review and meta-analysis. Clin. Rehabil. 2021;**35**(12):1661-73 doi: 10.1177/02692155211011944 [published Online First: 20210426].

23. Wang C, Zhang Z, Ma Y, Liu X, Zhu Q. Platelet-rich plasma injection vs corticosteroid injection for conservative treatment of rotator cuff lesions: A systematic review and meta-analysis. Medicine (Baltimore) 2021;**100**(7):e24680 doi: 10.1097/md.0000000000024680.

24. Peng Y, Li F, Ding Y, et al. Comparison of the effects of platelet-rich plasma and corticosteroid injection in rotator cuff disease treatment: a systematic review and meta-analysis. J. Shoulder Elbow Surg. 2023;**32**(6):1303-13 doi: 10.1016/j.jse.2023.01.037 [published Online First: 20230302].

25. Pang L, Xu Y, Li T, Li Y, Zhu J, Tang X. Platelet-Rich Plasma Injection Can Be a Viable Alternative to Corticosteroid Injection for Conservative Treatment of Rotator Cuff Disease: A Meta-analysis of Randomized Controlled Trials. Arthroscopy 2023;**39**(2):402-21.e1 doi: 10.1016/j.arthro.2022.06.022 [published Online First: 20220708].

26. MS AH, Sazlina SG. Platelet-rich plasma for rotator cuff tendinopathy: A systematic review and meta-analysis. PLoS One 2021;**16**(5):e0251111 doi: 10.1371/journal.pone.0251111 [published Online First: 20210510].

27. Lin M-T, Wei K-C, Wu C-H. Effectiveness of Platelet-Rich Plasma Injection in Rotator Cuff Tendinopathy: A Systematic Review and Meta-Analysis of Randomized Controlled Trials. Diagnostics 2020;**10**(4) doi: 10.3390/diagnostics10040189.

28. Hurley ET, Hannon CP, Pauzenberger L, Fat DL, Moran CJ, Mullett H. Nonoperative Treatment of Rotator Cuff Disease With Platelet-Rich Plasma: A Systematic Review of Randomized Controlled Trials. Arthroscopy 2019;**35**(5):1584-91 doi: 10.1016/j.arthro.2018.10.115 [published Online First: 20190415].
