# Supplementary material for: Differences in the effectiveness of leukocyte-rich platelet-rich plasma compared with leukocyte-poor platelet-rich plasma in the treatment of rotator cuff surgery: an umbrella review of meta-analyses
Source: J Orthop Traumatol. 2024 Oct 24;25:50. doi: 10.1186/s10195-024-00791-1 (PMC11502652; doi:10.1186/s10195-024-00791-1)
Supplement: Supplementary file 4 — Additional file 4. [file 10195_2024_791_MOESM4_ESM.docx]

**Table.** Quality evaluation of included studies based on AMSTAR.

| Study | 1. | 2. | 3. | 4. | 5. | 6. | 7. | 8. | 9. | 10. | 11. | 12. | 13. | 14. | 15. | 16. | Overall rating |
| --- | --- | --- | --- | --- | --- | --- | --- | --- | --- | --- | --- | --- | --- | --- | --- | --- | --- |
| Feltri P, ^1^ | Y | Y | Y | Y | Y | Y | Y | Y | Y | N | Y | Y | N | Y | Y | Y | Moderate |
| Li Y ,^2^ | Y | Y | Y | Y | Y | Y | Y | Y | Y | Y | Y | Y | Y | Y | Y | Y | High |
| Zhao D,^3^ | Y | Y | Y | Y | Y | Y | Y | Y | Y | N | Y | N | Y | Y | Y | Y | Moderate |
| Xu W, ^4^ | Y | Y | Y | Y | Y | Y | Y | Y | Y | N | Y | Y | N | Y | Y | Y | Moderate |
| Ryan J,^5^ | Y | Y | Y | Y | Y | Y | Y | Y | Y | Y | Y | Y | Y | Y | Y | Y | High |
| Yang FA,^6^ | Y | Y | Y | Y | Y | Y | Y | N | Y | N | Y | Y | Y | Y | Y | Y | Moderate |
| Chen X,^7^ | Y | Y | Y | Y | Y | Y | Y | Y | Y | Y | Y | Y | N | Y | Y | Y | Moderate |
| Wang C, ^8^ | Y | Y | Y | Y | Y | Y | Y | Y | Y | Y | Y | Y | Y | Y | Y | Y | High |
| Han C, ^9^ | Y | Y | Y | Y | Y | Y | Y | Y | Y | Y | Y | Y | Y | Y | Y | Y | High |
| Zhao JG, ^10^ | Y | Y | Y | Y | Y | Y | Y | Y | Y | N | Y | Y | Y | N | Y | Y | Moderate |
| Cai YZ, ^11^ | Y | Y | Y | Y | Y | N | Y | Y | Y | Y | Y | Y | N | Y | Y | Y | Moderate |

Scale of item score: N, no; Y, yes.

The AMSTAR criteria are Q1: Did the research questions and inclusion criteria for the review include the components of PICO?, Q2: Did the report of the review contain an explicit statement that the review methods were established prior to the conduct of the review and did the report justify any significant deviations from the protocol?, Q3: Did the review authors explain their selection of the study designs for inclusion in the review?, Q4: Did the review authors use a comprehensive literature search strategy?, Q5: Did the review authors perform study selection in duplicate?, Q6: Did the review authors perform data extraction in duplicate?, Q7: Did the review authors provide a list of excluded studies and justify the exclusions?, Q8: Did the review authors describe the included studies in adequate detail?, Q9: Did the review authors use a satisfactory technique for assessing the risk of bias (RoB) in individual studies that were included in the review?, Q10: Did the review authors report on the sources of funding for the studies included in the review?, Q11: If meta-analysis was performed, did the review authors use appropriate methods for statistical combination of results?, Q12: If meta-analysis was performed, did the review authors assess the potential impact of RoB in individual studies on the results of the meta-analysis or other evidence synthesis?, Q13: Did the review authors account for RoB in primary studies when interpreting/discussing the results of the review?, Q14: Did the review authors provide a satisfactory explanation for, and discussion of, any heterogeneity observed in the results of the review?, Q15: If they performed quantitative synthesis did the review authors carry out an adequate investigation of publication bias (small study bias) and discuss its likely impact on the results of the review?, Q16: Did the review authors report any potential sources of conflict of interest, including any funding they received for conducting the review?

## Reference：

**1.** Feltri P, Gonalba GC, Boffa A, et al. Platelet-rich plasma does not improve clinical results in patients with rotator cuff disorders but reduces the retear rate. A systematic review and meta-analysis. *Knee Surg Sports Traumatol Arthrosc.* 2023;31:1940-1952.

**2.** Li Y, Li T, Li J, Tang X, Li R, Xiong Y. Platelet-Rich Plasma Has Better Results for Retear Rate, Pain, and Outcome Than Platelet-Rich Fibrin After Rotator Cuff Repair: A Systematic Review and Meta-analysis of Randomized Controlled Trials. *Arthroscopy.* 2022;38:539-550.

**3.** Zhao D, Han Y-H, Pan J-K, et al. The clinical efficacy of leukocyte-poor plateletrich plasma in arthroscopic rotator cuff repair: a meta-analysis of randomized controlled trials. *J Shoulder Elbow Surg.* 2021;30:918-928.

**4.** Xu W, Xue Q. Application of Platelet-Rich Plasma in Arthroscopic Rotator Cuff Repair: A Systematic Review and Meta-analysis. *Orthopaedic Journal of Sports Medicine.* 2021;9.

**5.** Ryan J, Imbergamo C, Sudah S, et al. Platelet-Rich Product Supplementation in Rotator Cuff Repair Reduces Retear Rates and Improves Clinical Outcomes: A Meta-analysis of Randomized Controlled Trials. *Arthroscopy.* 2021;37:2608-2624.

**6.** Yang FA, Liao CD, Wu CW, Shih YC, Wu LC, Chen HC. Effects of applying platelet-rich plasma during arthroscopic rotator cuff repair: a systematic review and meta-analysis of randomised controlled trials. *Sci Rep.* 2020;10:17171.

**7.** Chen X, Jones IA, Togashi R, Park C, Vangsness CT, Jr. Use of Platelet-Rich Plasma for the Improvement of Pain and Function in Rotator Cuff Tears: A Systematic Review and Meta-analysis With Bias Assessment. *Am J Sports Med.* 2020;48:2028-2041.

**8.** Wang C, Xu M, Guo W, Wang Y, Zhao S, Zhong L. Clinical efficacy and safety of platelet-rich plasma in arthroscopic full-thickness rotator cuff repair: A meta-analysis. *PLoS One.* 2019;14:e0220392.

**9.** Han C, Na Y, Zhu Y, et al. Is platelet-rich plasma an ideal biomaterial for arthroscopic rotator cuff repair? A systematic review and meta-analysis of randomized controlled trials. *J Orthop Surg Res.* 2019;14:183.

**10.** Zhao JG, Zhao L, Jiang YX, Wang ZL, Wang J, Zhang P. Platelet-rich plasma in arthroscopic rotator cuff repair: a meta-analysis of randomized controlled trials. *Arthroscopy.* 2015;31:125-135.

**11.** Cai YZ, Zhang C, Lin XJ. Efficacy of platelet-rich plasma in arthroscopic repair of full-thickness rotator cuff tears: a meta-analysis. *J Shoulder Elbow Surg.* 2015;24:1852-1859.
