# Supplementary material for: Differences in the effectiveness of leukocyte-rich platelet-rich plasma compared with leukocyte-poor platelet-rich plasma in the treatment of rotator cuff surgery: an umbrella review of meta-analyses
Source: J Orthop Traumatol. 2024 Oct 24;25:50. doi: 10.1186/s10195-024-00791-1 (PMC11502652; doi:10.1186/s10195-024-00791-1)
Supplement: Supplementary file 5 — Additional file 5. [file 10195_2024_791_MOESM5_ESM.docx]

**Supplementary material Table 1:** Results of meta-analysis of Retear Rates in Leukocyte-Poor Platelet-Rich Plasma group.

| **Study** | **Year** | **Type of metric (summary effect)** | **Effect** | **95% CI** | **Number of included studies** | **I²** | **P** |
| --- | --- | --- | --- | --- | --- | --- | --- |
| Li Y[1] | 2022 | RR | 0.52 | (0.37 , 0.72) | 9 | 39 | <0.01 |
| Zhao D[2] | 2021 | RR | 0.59 | (0.45 , 0.77) | 8 | 0 | <0.01 |
| Xu W[3] | 2021 | RR | 0.62 | (0.42 , 0.92) | 9 | 19 | 0.02 |
| Yang FA[4] | 2020 | RR | 0.37 | (0.21 , 0.65) | 6 | 0 | <0.01 |
| Zhao JG[5] | 2015 | RR | 1.12 | ( 0.80 , 1.56) | 5 | 46 | 0.52 |

**Supplementary material Table 2:** Results of meta-analysis of Constant Score in Leukocyte-Poor Platelet-Rich Plasma group.

| **Study** | **Year** | **Type of metric (summary effect)** | **Effect** | **95% CI** | **Number of included studies** | **I²** | **P** |
| --- | --- | --- | --- | --- | --- | --- | --- |
| Li Y[1] | 2022 | MD | 2.53 | (0.93 , 4.13) | 9 | 0 | 0.02 |
| Zhao D[2] | 2021 | MD | 3.35 | (1.68 , 5.02) | 7 | 19 | <0.01 |
| Ryan J[6] | 2021 | MD | 2.11 | （-0.06 , 4.29) | 8 | 44 | 0.05 |
| Yang FA[4] | 2020 | MD | -0.10 | (-4.35 , 4.15) | 3 | 0 | 0.96 |
| Han C[7] | 2019 | MD | 2.57 | (0.81 , 4.34) | 6 | 0 | <0.01 |
| Zhao JG[5] | 2015 | MD | 3.24 | (-3.76 , 10.24) | 2 | 0 | 0.36 |
| Cai YZ[8] | 2015 | MD | -1.30 | (-6.20 , 3.60) | 2 | 0 | 0.60 |

**Supplementary material Table 3:** Results of meta-analysis of University of California, Los Angeles score in Leukocyte-Poor Platelet-Rich Plasma group.

| **Study** | **Year** | **Type of metric (summary effect)** | **Effect** | **95% CI** | **Number of included studies** | **I²** | **P** |
| --- | --- | --- | --- | --- | --- | --- | --- |
| Li Y[1] | 2022 | MD | 0.86 | (-0.41 , 2.13) | 6 | 54 | 0.18 |
| Zhao D[2] | 2021 | MD | 1.73 | (0.94 , 2.52) | 5 | 49 | <0.01 |
| Ryan J[6] | 2021 | MD | 0.51 | (-0.94 , 1.97) | 6 | 66 | 0.50 |
| Yang FA[4] | 2020 | MD | -0.32 | (-1.89, 1.24) | 3 | 0 | 0.68 |
| Han C[7] | 2019 | MD | 0.78 | (-0.47 ,2.02) | 6 | 54 | 0.22 |
| Zhao JG[5] | 2015 | MD | –0.93 | (–2.44 , 0.57) | 3 | 0 | 0.22 |
| Cai YZ[8] | 2015 | MD | 0.44 | (-2.10 , 2.98) | 3 | 73 | 0.73 |

**Supplementary material Table 4:** Results of meta-analysis of Visual Analog Scale in Leukocyte-Poor Platelet-Rich Plasma group.

| **Study** | **Year** | **Type of metric (summary effect)** | **Effect** | **95% CI** | **Number of included studies** | **I²** | **P** |
| --- | --- | --- | --- | --- | --- | --- | --- |
| Li Y[1] | 2022 | MD | -0.11 | (-0.18 , -0.04) | 8 | 0 | <0.01 |
| Zhao D[2] | 2021 | MD | -0.31 | (-0.44 , -0.18) | 7 | 21 | <0.01 |
| Ryan J[6] | 2021 | MD | -0.19 | (-0.36 , -0.03) | 4 | 0 | 0.02 |
| Yang FA[4] | 2020 | MD | -0.16 | (-0.33 , 0.01) | 3 | 0 | 0.06 |
| Han C[7] | 2019 | MD | -0.20 | (-0.36 , -0.04) | 4 | 0 | 0.01 |

**Supplementary material Table 5:** Results of meta-analysis of American Shoulder and Elbow Surgeons score in Leukocyte-Poor Platelet-Rich Plasma group.

| **Study** | **Year** | **Type of metric (summary effect)** | **Effect** | **95% CI** | **Number of included studies** | **I²** | **P** |
| --- | --- | --- | --- | --- | --- | --- | --- |
| Li Y[1] | 2022 | MD | 1.75 | (-0.17 , 3.66) | 5 | 0 | 0.07 |
| Zhao D[2] | 2021 | MD | 1.75 | (-0.13 , 3.63) | 5 | 0 | 0.07 |
| Ryan J[6] | 2021 | MD | 0.37 | (-2.20 , 2.95） | 6 | 41 | 0.78 |
| Han C[7] | 2019 | MD | 0.90 | (-0.77 , 2.57) | 7 | 26 | 0.29 |
| Cai YZ[8] | 2015 | MD | 0.91 | (-3.72 , 5.54) | 2 | 0 | 0.70 |

**Supplementary material Table 6:** Results of meta-analysis of Simple Shoulder Test in Leukocyte-Poor Platelet-Rich Plasma group.

| **Study** | **Year** | **Type of metric (summary effect)** | **Effect** | **95% CI** | **Number of included studies** | **I²** | **P** |
| --- | --- | --- | --- | --- | --- | --- | --- |
| Ryan J[6] | 2021 | MD | 0.47 | (-0.36 , 1.30) | 2 | 0 | 0.26 |
| Han C[7] | 2019 | MD | 0.47 | (-0.36 , 1.30) | 2 | 0 | 0.26 |

**Supplementary material Table 7:** Results of meta-analysis of Retear Rates in Leukocyte-Rich Platelet-Rich Plasma group.

| **Study** | **Year** | **Type of metric (summary effect)** | **Effect** | **95% CI** | **Number of included studies** | **I²** | **P** |
| --- | --- | --- | --- | --- | --- | --- | --- |
| Li Y[1] | 2022 | RR | 0.66 | (0.41 , 1.07) | 3 | 0 | 0.10 |
| Xu W[3] | 2021 | RR | 0.69 | (0.42 , 1.11) | 3 | 0 | 0.12 |
| Zhao JG[5] | 2015 | RR | 0.63 | ( 0.34 , 1.19) | 2 | 33 | 0.22 |

**Supplementary material Table 8:** Results of meta-analysis of Constant Score in Leukocyte-Rich Platelet-Rich Plasma group.

| **Study** | **Year** | **Type of metric (summary effect)** | **Effect** | **95% CI** | **Number of included studies** | **I²** | **P** |
| --- | --- | --- | --- | --- | --- | --- | --- |
| Li Y[1] | 2022 | MD | 1.96 | (-0.55 , 4.47) | 4 | 0 | 0.13 |
| Ryan J[6] | 2021 | MD | 3.44 | (1.12 , 5.76) | 2 | 0 | <0.01 |
| Han C[7] | 2019 | MD | 2.98 | （1.01 , 4.94) | 3 | 0 | <0.01 |

**Supplementary material Table 9:** Results of meta-analysis of Simple Shoulder Test in Leukocyte-Rich Platelet-Rich Plasma group.

| **Study** | **Year** | **Type of metric (summary effect)** | **Effect** | **95% CI** | **Number of included studies** | **I²** | **P** |
| --- | --- | --- | --- | --- | --- | --- | --- |
| Ryan J[6] | 2021 | MD | 0.40 | (0.05 , 0.75) | 2 | 0 | 0.02 |
| Han C[7] | 2019 | MD | 0.43 | (0.08 , 0.78) | 2 | 0 | 0.02 |

**Reference:**

1. Li Y, Li T, Li J, Tang X, Li R, Xiong Y. Platelet-Rich Plasma Has Better Results for Retear Rate, Pain, and Outcome Than Platelet-Rich Fibrin After Rotator Cuff Repair: A Systematic Review and Meta-analysis of Randomized Controlled Trials. Arthroscopy 2022;**38**(2):539-50 doi: 10.1016/j.arthro.2021.05.023 [published Online First: 20210527].

2. Zhao D, Han Y-H, Pan J-K, et al. The clinical efficacy of leukocyte-poor plateletrich plasma in arthroscopic rotator cuff repair: a meta-analysis of randomized controlled trials. J. Shoulder Elbow Surg. 2021;**30**(4):918-28 doi: 10.1016/j.jse.2020.10.014.

3. Xu W, Xue Q. Application of Platelet-Rich Plasma in Arthroscopic Rotator Cuff Repair: A Systematic Review and Meta-analysis. Orthopaedic Journal of Sports Medicine 2021;**9**(7) doi: 10.1177/23259671211016847.

4. Yang FA, Liao CD, Wu CW, Shih YC, Wu LC, Chen HC. Effects of applying platelet-rich plasma during arthroscopic rotator cuff repair: a systematic review and meta-analysis of randomised controlled trials. Sci. Rep. 2020;**10**(1):17171 doi: 10.1038/s41598-020-74341-0 [published Online First: 20201014].

5. Zhao JG, Zhao L, Jiang YX, Wang ZL, Wang J, Zhang P. Platelet-rich plasma in arthroscopic rotator cuff repair: a meta-analysis of randomized controlled trials. Arthroscopy 2015;**31**(1):125-35 doi: 10.1016/j.arthro.2014.08.008 [published Online First: 20140930].

6. Ryan J, Imbergamo C, Sudah S, et al. Platelet-Rich Product Supplementation in Rotator Cuff Repair Reduces Retear Rates and Improves Clinical Outcomes: A Meta-analysis of Randomized Controlled Trials. Arthroscopy 2021;**37**(8):2608-24 doi: 10.1016/j.arthro.2021.03.010 [published Online First: 20210317].

7. Han C, Na Y, Zhu Y, et al. Is platelet-rich plasma an ideal biomaterial for arthroscopic rotator cuff repair? A systematic review and meta-analysis of randomized controlled trials. J. Orthop. Surg. Res. 2019;**14**(1):183 doi: 10.1186/s13018-019-1207-9 [published Online First: 20190620].

8. Cai YZ, Zhang C, Lin XJ. Efficacy of platelet-rich plasma in arthroscopic repair of full-thickness rotator cuff tears: a meta-analysis. J. Shoulder Elbow Surg. 2015;**24**(12):1852-9 doi: 10.1016/j.jse.2015.07.035 [published Online First: 20151009].
