# Supplementary material for: Differences in the effectiveness of leukocyte-rich platelet-rich plasma compared with leukocyte-poor platelet-rich plasma in the treatment of rotator cuff surgery: an umbrella review of meta-analyses
Source: J Orthop Traumatol. 2024 Oct 24;25:50. doi: 10.1186/s10195-024-00791-1 (PMC11502652; doi:10.1186/s10195-024-00791-1)
Supplement: Supplementary file 6 — Additional file 6. [file 10195_2024_791_MOESM6_ESM.docx]

# 1.

Pubmed

Platelet-Rich Plasma

Rotator Cuff

Meta-analysis

Systematic review

# 2.

## Embase

meta analysis

systematic review

rotator cuff

plasma, platelet-rich

# 3.

## Web of science

Platelet-rich Plasma

Rotator Cuff

Systematic review

Meta-analysis

# 4.

## Cochrane

meta analysis

systematic review

Rotator Cuff

Platelet-Rich Plasma
